# Application of mobile-technology for disease and treatment monitoring of malaria in the "Better Border Healthcare Programme"

**DOI:** 10.1186/1475-2875-9-237

**Published:** 2010-08-19

**Authors:** Pongthep Meankaew, Jaranit Kaewkungwal, Amnat Khamsiriwatchara, Podjadeach Khunthong, Pratap Singhasivanon, Wichai Satimai

**Affiliations:** 1 Center of Excellence for Biomedical and Public Health Informatics (BIOPHICS), Faculty of Tropical Medicine, Mahidol University, Bangkok, Thailand; 2 Department of Tropical Hygiene, Faculty of Tropical Medicine, Mahidol University, Bangkok, Thailand; 3 Bureau of Vector-borne Diseases, Department of Disease Control, Ministry of Public Health, Nonthaburi, Thailand

## Abstract

**Background:**

The main objective of this study was to assess the effectiveness of integrating the use of cell-phones into a routine malaria prevention and control programme, to improve the management of malaria cases among an under-served population in a border area. The module for disease and treatment monitoring of malaria (DTMM) consisted of case investigation and case follow-up for treatment compliance and patients' symptoms.

**Methods:**

The module combining web-based and mobile technologies was developed as a proof of concept, in an attempt to replace the existing manual, paper-based activities that malaria staff used in treating and caring for malaria patients in the villages for which they were responsible. After a patient was detected and registered onto the system, case-investigation and treatment details were recorded into the malaria database. A follow-up schedule was generated, and the patient's status was updated when the malaria staff conducted their routine home visits, using mobile phones loaded with the follow-up application module. The module also generated text and graph messages for a summary of malaria cases and basic statistics, and automatically fed to predetermined malaria personnel for situation analysis. Following standard public-health practices, access to the patient database was strictly limited to authorized personnel in charge of patient case management.

**Results:**

The DTMM module was developed and implemented at the trial site in late November 2008, and was fully functioning in 2009. The system captured 534 malaria patients in 2009. Compared to paper-based data in 2004-2008, the mobile-phone-based case follow-up rates by malaria staff improved significantly. The follow-up rates for both Thai and migrant patients were about 94-99% on Day 7 *(Plasmodium falciparum) *and Day 14 *(Plasmodium vivax) *and maintained at 84-93% on Day 90. Adherence to anti-malarial drug therapy, based on self-reporting, showed high completion rate for *P. falciparum*-infected cases, but lower rate for *P. vivax *cases. Patients' symptoms were captured onto the mobile phone during each follow-up visit, either during the home visit or at Malaria Clinic; most patients had headache, muscle pain, and fatigue, and some had fever within the first follow-up day (day7/14) after the first anti-malarial drug dose.

**Conclusions:**

The module was successfully integrated and functioned as part of the malaria prevention and control programme. Despite the bias inherent in sensitizing malaria workers to perform active case follow-up using the mobile device, the study proved for its feasibility and the extent to which community healthcare personnel in the low resource settings could potentially utilize it efficiently to perform routine duties, even in remote areas. The DTMM has been modified and is currently functioning in seven provinces in a project supported by the WHO and the Bill & Melinda Gates Foundation, to contain multi-drug resistant malaria on the Thai-Cambodian border.

## Background

The World Health Organization (WHO) has set ultimate goal to fight against malaria towards the elimination of the disease by which starts with good and effective malaria control programme [1,2]. Several control measures and interventions have been developed and implemented across the region, including mosquito control, indoor residual spraying, insecticide-treated nets, prompt and effective treatment, intermittent preventive prophylaxis, and behavioural change education [3,4]. One of the key strategies for eliminating malaria is the prompt identification and treatment of malaria patients. To achieve this goal, an effective disease-management system should exist to enable rapid and accurate malaria case detection in target areas, and ensure effective treatment. Therefore, an effective system should allow case detection for early treatment at the point-of-care, and real-time case investigation and active follow-up of positive cases at the community level [5,6]. Although the vertical control programme administered by the Bureau of Vector-borne Diseases (BVBD) in Thailand is well organized and distributed throughout epidemic areas across the country, preventive and treatment control measures may not cover fully and efficiently in rural and remote communities. Difficulties in those communities might include limited service access, due to poverty and illegal immigration status, which inhibit villagers' treatment-seeking behaviours [7].

In the BVBD's vertical malaria-control programme [8], malaria-case treatment and care are managed down to the village level. In villages in epidemic areas, village malaria volunteers (VMVs) and/or village health volunteers (VHVs) work at the point-of-care, malaria posts (MPs), which collaborate with staff at the official malaria clinic (MC). At the upper levels, treatment and care are monitored vertically by the Vector-borne Disease Unit (VBDU), Vector-borne Disease Center (VBDC), up to the regional Offices of Disease Prevention and Control (ODPC), and BVBD. In passive case detection, the local febrile patients visit at MC/MP or VBDU office for diagnosis. The VBDU staff, however, also conduct periodic active case detection in their responsible villages. The infected cases are registered and investigated in more detail. According to the standard malaria case management practices of Thailand's Ministry of Public Health (MOPH), medication and follow-up days are different, corresponding with the type of infection. The first-line drugs for malaria treatment adopted by the MOPH adhere to the national guidelines of Thailand and the WHO [9-13]. Typical uncomplicated *Plasmodium falciparum *cases are treated with three days of artesunate, plus mefloquine on day2 and day3; with recommended follow-up at days 7 and 28, with additional days 60 and 90 [13-16]. For typical *Plasmodium vivax *malaria cases, patients are treated according to recommended WHO and Thai MOPH practices, with chloroquine for three days and a 14-day course of primaquine to prevent relapse; with recommended follow-up on days 14, 28, 60, and 90 [14,17-19].

A major challenge for malaria-control programme is the assurance of prompt and effective treatment. It has been suggested in the literature that monitoring of prompt diagnosis and effective management of acute clinical episodes with anti-malarial drugs are crucial to reducing morbidity and mortality [20-22]. The WHO suggests that standard guidelines on duration of follow-up for safety monitoring are needed in different regions [11,23]. In Thailand, designated malaria staff routinely assess safety and tolerability, and monitor clinical failures, in accord with WHO guidelines. Strategies to promote patient adherence would improve drug performance and thereby might help to prevent the rapid emergence of drug resistance [24,25].

This study was part of the project *Application of Smart Phone in "Better Border Healthcare Programme" (BBHP) *that was awarded by the Microsoft Research in early 2008. The main objective of this two-year project was to develop technology-based healthcare solutions that would increase the accessibility and affordability of treatment and care services to the under-served communities [26]. The BBHP modules were developed in part to correspond with the United Nations' Millennium Development Goals (MDG), and BVBD malarial-control indicators. One MDG targeting healthcare services included combating malaria and other diseases, while the BVBD had set its mission for prevention and control of malaria to eventually eliminate it. The module for Disease and Treatment Monitoring of Malaria (DTMM), one part of the BBHP project, was thus developed in an attempt to find a solution using mobile technology to alter treatment-seeking behaviours and facilitate better treatment and care for malaria patients in low-resource settings. Mobile technology, particularly the cellular phone, has not only penetrated the daily lives of people in metropolitan areas and large rural cities/towns; they have also become popular among those living in remote areas. Thus the DTMM combined both web-based and smart-phone applications to manage malaria treatment and care activities, integrated into routine case detection and follow-up at selected MCs and VBDU. The main objective of this study was to assess the module's effectiveness in improving case follow-up and treatment compliance among an under-served population in a border area.

## Methods

### Setting and study population

The DTMM was implemented in pilot areas of Sai Yok District, western Kanchanaburi Province, along the Thai-Myanmar border. The district, with an area of 2,728.922 sq km (1,053.6 sq mi), is divided into seven sub-districts, including 55 villages; there are 11 health centers, two sub-district hospitals, two VBDUs, and three MCs in the district [27,28]. The DTMM study areas covered three sub-districts (Bongti, Sri Monkol, and Lum-Sum), 13 main villages, plus some hamlets of other nearby villages, from which inhabitants sought treatment under the treatment coverage area of one MC and one VBDU. Most residents in the area are of Thai, Karen, Mon, and Myanmar ethnicity, and are farmers, woodcutters, and laborers. The non-Thai or migrants were classified as M1, or migrant workers who had been living or working in Thailand ≥ 6 months; and M2, or migrant workers who had been living or working in Thailand < 6 months.

At-risk groups for malaria transmission in the study area included Thais and migrants who resided along the border, and also uncontrolled and undocumented cross-border migrants. The latter group also sought treatment from the MP/MC in the area [29,30]. According to the BVBD stratification system [7], the types of transmission area are divided by control indicators; A1 (perennial malaria transmission area) is an area where transmission is reported ≥ 6 months per year; A2 (periodic malaria transmission area) is an area where transmission is reported ≤ 5 months per year; B1 (high-risk area) is where transmission has not been reported within the last three years, but primary and secondary vectors are found, and consequently, the area is potentially suitable for malaria transmission (high and moderate receptivity); B2 (low risk area) is where transmission has not been reported within the last three years; neither primary nor secondary vectors are found, and suspected vectors may be found (low and non-receptive).

Sai Yok District is located in a region with a tropical climate, typically with a rainy season in May-November, when most malaria cases are present; however, cases have been reported throughout the year in this malaria-transmission zone. The three sub-districts where the DTMM was implemented, were classified as A1 areas. The annual parasite incidence (API) of malaria in Sai Yok District in 2009 was 17.3/1,000 population; Bongtee sub-district had the highest API, of 126.1/1,000 population [31,32]. It was reported that *P. falciparum *and *P. vivax *were present in roughly equal proportions [31].

### DTMM module

**Figure 1:**
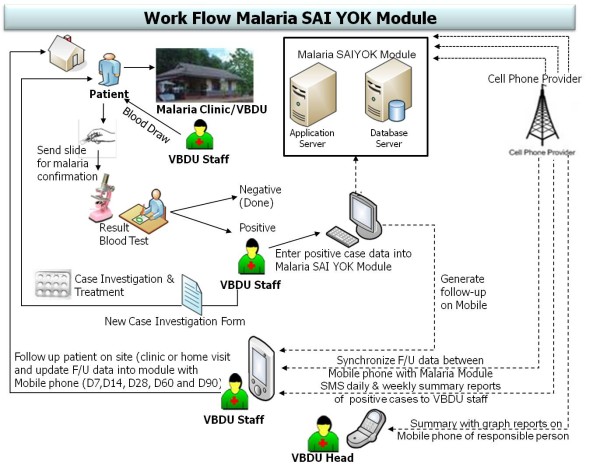
**Conceptual framework and work flow of DTMM**.

The DTMM was developed under the standard software development life cycle (SDLC) approach to cover malaria case management at the local community MCs and VBDU. This innovative module was designed to be consistent with the existing paper-based workflow to avoid resistance from changing the ways in which malaria staff performed their routine treatment and care activities. To make it more feasible for effective utilization in a field setting, some new features were added to the module while some redundant parts related to data collection were eliminated. The DTMM adopted mobile technology-based features to replace the standard manual paper-based methods of case detection and follow-up in the villages. Figure 1 shows the conceptual framework and workflow of the DTMM.

**Figure 2:**
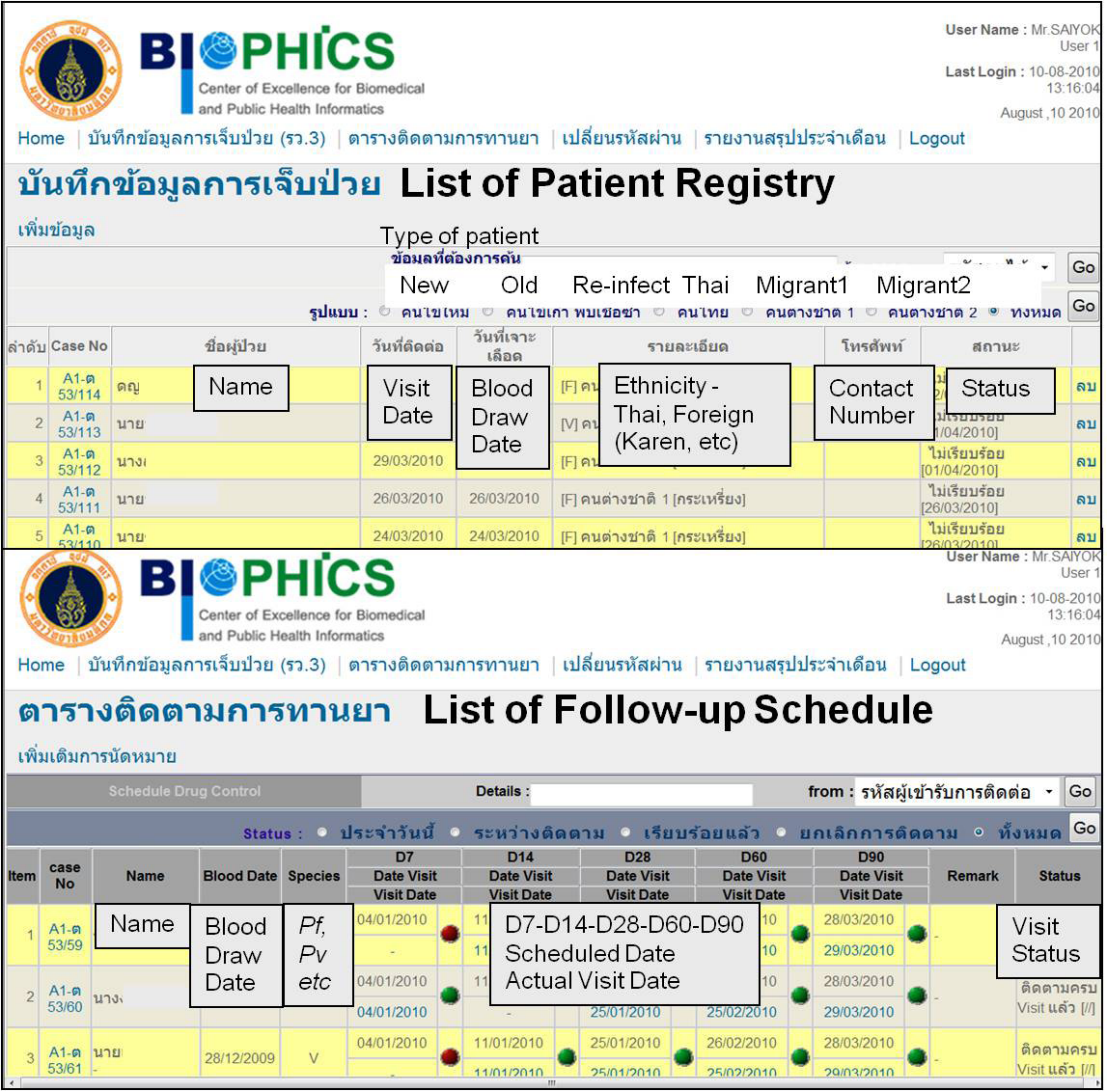
**Screen shots of case registry and follow-up schedule**.

The three main functions of the DTMM are case detection/registry, new case investigation, and case follow-up. The case detection/registry and investigation functions of the DTMM have been adapted from the standard paper-based data collection of the infected patients. In the case-investigation form, details of case characteristics, type of malaria, and treatment, are collected. After a patient receives medication per standard treatment guidelines, a follow-up schedule is generated and updated each time follow-up is performed. Once the data have been entered into the module, each individual case, or list of registered or followed-up patients in the system, who have visited the MC/VBDU, can be examined by the responsible staff (see Figure 2).

**Figure 3:**
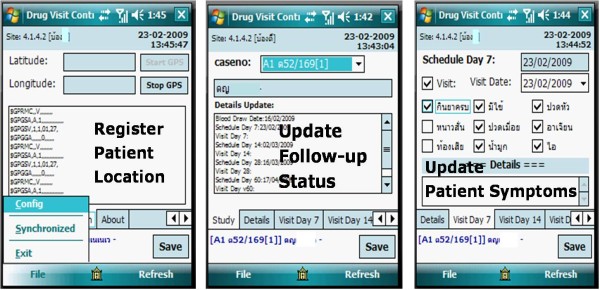
**Screen shots of case follow-up on mobile phone**.

Responsible malaria staff at the local treatment sites are provided with mobile phones loaded with the follow-up application module; they use the case-follow-up function of the DTMM to update the follow-up status on the schedule date, and capture the locations (coordinates) each time local malaria staff perform routine home visits. This replaces paper-based case-tracking in the villages, and supersedes routine map-drawing for case locations. The DTMM also sends home-visit-schedule reminder messages directly to responsible local staff. During a home visit, the DTMM follow-up function can record the typical clinical symptoms of malaria. Information can be captured on the phone in areas without a telephone signal, and malaria-control staff can later synchronize information onto the system at the MC/VBDU, or wherever a signal is available (see Figure 3).

**Figure 4:**
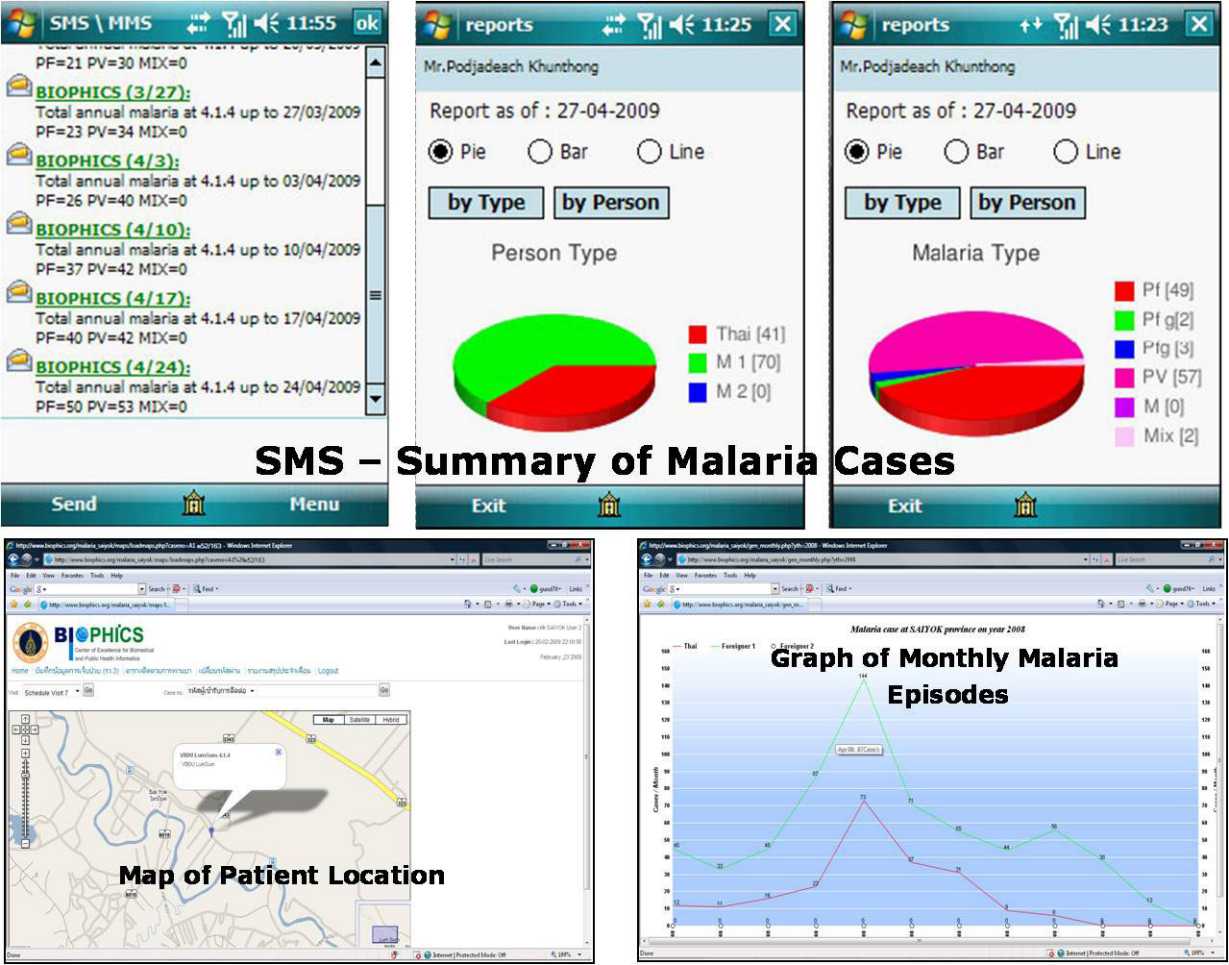
**Screen shots of summary reports & graphs on mobile phone/site workstation**.

The use of the DTMM allows remote data transfer technology in both textual and geographic format. On a weekly basis, the system would generate short message service (SMS) with a summary of malaria cases, and automatically feed them to predetermined MC/VBDU personnel. A map of each scheduled visit can be displayed by clicking on the visit schedule table; this helps to locate cases in the area, and is especially useful for identifying foreign cases in remote border areas. Maps of all cases covered by the health service areas may be seen at the MC, and also at the upper supervisory level. Summary statistics can be generated to help malaria authorities make informed decisions and act accordingly (see Figure 4).

### Ethical considerations

It should be noted that all patient-management activities and the database containing information associated with such activities, were strictly secured and used only by authorized personnel who were in charge of patient case-management. The electronic DTMM system maintains the same crucial data-integrity and confidentiality features as the paper-based processes at the local malaria clinics.

No written informed consent forms were signed by the patients who visited the malaria clinics, since the activities were all routine malaria clinic work; however, the staff had verbally informed the patients and asked them to return to the clinic, or agree to home visits, as part of their scheduled follow-up. Data extracted from the DTMM databases were secondary data with no identification linked to them. The authors requested official permission from the BVBD Director to use the extracted data for analysis. This study protocol was reviewed and approved by the Ethics Committee of the Faculty of Topical Medicine, Mahidol University. Electronic data were encrypted for transfer, to ensure confidentiality and security.

## Results

### Malaria case investigation

The DTMM module was developed and implemented at the trial site in late November 2008 and was fully functioning in 2009. During 2009, 534 malaria patients, 324 (61%) Thais, and 210 (39%) migrants, visited the two malaria clinics in Sai Yok area (Table 1). Almost all of the migrants were Karen and other minority groups who resided in Thailand for ≥ 6 months (M1). The percentage of patients with *P. falciparum *(56%) appeared to be slightly higher than *P. vivax *(44%) among the Thais, while the percentages of *P. falciparum *and *P. vivax *were about the same among the migrants. Some cases had a history of infection, and used to visit the MCs (classified by the local malaria staff as "not cured" or "re-infected" cases); the percentage of old cases among the migrants (15.3%) was higher than the Thais (7.1%).

**Table 1:** Case investigation of malaria patients in Sai Yok District, 2009

Characteristics	Migrant (n = 210)		Thai (n = 324)	
	n	%	n	%
Type of Infection				
Species				
*Plasmodium falciparum*	104	49.5	181	55.9
*Plasmodium vivax*	106	50.5	143	44.1
Type of patient				
New case	178	84.8	301	92.9
Old case - not cured	6	2.9	4	1.2
Old case - reinfection	26	12.4	19	5.9
**Personal characteristics**				
Gender				
Male	151	71.9	235	72.5
Female	59	28.1	89	27.5
Age Group (Year)				
< = 10	19	9.0	91	28.1
11-20	57	27.1	89	27.5
21-30	30	14.3	59	18.2
31-40	35	16.7	45	13.9
41-50	36	17.1	19	5.9
51-60	22	10.5	17	5.2
> = 61	11	5.2	4	1.2
Occupation				
Pre-school child	10	4.8	35	10.8
Student	32	15.2	90	27.8
Agricultural - farming	131	62.4	172	53.1
Work in forest - cut wood, hunting	22	10.5	17	5.2
Commercial	8	3.8	3	0.9
Labor	4	1.9	7	2.2
Other	3	1.4	0	0.0
Marital status				
Not married	90	42.9	207	63.9
Married	120	57.1	117	35.1
**Risk factors**				
Stayed out of household (before infection)				
No	87	41.4	111	34.3
Yes - within town/province	99	47.1	95	29.3
Yes - in Myanmar	24	11.4	116	35.8
Yes - not specified	0	0.0	2	0.6
Own bed-net				
Not own any bed-net	7	3.3	7	2.2
Own at least 1 bed-net	203	96.7	317	97.8
Amount of bed-nets in household				
1	47	22.4	142	43.8
2-3	147	70.0	165	51.0
4-5	7	3.4	10	3.0
Sleep in bed-net when staying in transmission area				
No	64	30.5	75	23.1
Yes	143	68.1	246	75.9
Not specified	3	1,4	3	0.9
Staying in sprayed household				
No	98	46.7	154	47.5
Yes	109	51.9	168	51.9
Not specified	3	1.4	2	0.6

Over 70% of patients were male; most of them worked in rice and corn farms in the area. The average age of the Thai patients (Mean 22.1 ± SD 15.4; Median 19, Range 83) was significantly different from the migrants (Mean 31.3 ± SD 16.8; Median 30, Range 77). Among the Thais, more patients were children aged < 10 years (28%) than among the migrants (9%). By contrast, among the migrants, more patients were elderly than among the Thais.

Case investigation by malaria staff, attempting to explore possible risk factors based on self-reporting, found that 41% of migrants and 34% of Thais did not leave their households or current places of residence before experiencing febrile symptoms; these could be indigenous cases. Interestingly, self-reports of leaving the household 1-2 days before presumable infection, showed that more migrant patients stayed elsewhere in the province (47%) than Thais (29%), while more Thais (36%) traveled across the border than migrants (11%).

Almost everyone owned a bed-net to protect themselves from mosquito bite; over half of the families had > 1 bed-net. About 70% or more of both Thai and migrant patients reported that they usually slept in a bed-net when staying in a transmission area. About 50% of both groups reported that they stayed in a house that had been sprayed.

### Malaria case follow-up

Baseline data were collected for all malaria cases in the study area, for the years 2004-2008, before implementation of the DTMM (Table 2). The numbers of both Thai and migrant malaria patients varied; there appeared to be more migrant than Thai cases most years, and an outbreak was observed among migrants in 2008. Case follow-up percentages on Day 7 (for *P. falciparum*), Day 14 (for *P. vivax*), and Day 28 (both) during the period 2004-2008 also varied in the range 20-40% among the Thai cases, but could be < 10% among the migrants. The follow-up rates on Day 28 were usually lower than Day 7.

**Table 2:** Malaria case follow-up, 2004-2009

Year	*P. falciparum*			*P. vivax*		
	Total cases	% Follow-up		Total cases	% Follow-up	
		Day 7	Day 28		Day 14	Day 28
Thai						
2009	181	99.4	98.3	143	97.9	97.9
2008	161	36.7	26.1	110	33.6	26.4
2007	115	32.2	20.9	99	35.4	21.2
2006	133	38.4	22.6	104	24.0	12.5
2005	113	27.4	22.1	81	32.1	19.8
2004	209	36.4	30.1	186	37.1	37.1
Migrant						
2009	104	93.9	97.0	106	98.1	98.1
2008	444	25.2	5.6	231	25.1	6.1
2007	293	11.6	12.9	101	5.1	8.9
2006	312	17.9	9.3	106	27.4	13.2
2005	227	19.8	18.5	77	31.2	20.8
2004	205	30.7	32.7	167	35.3	28.7

**Figure 5:**
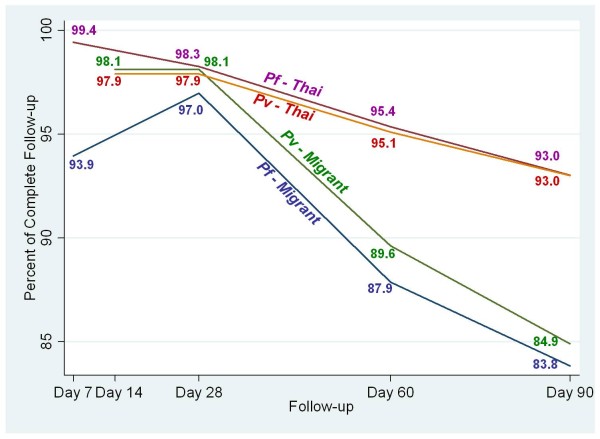
**Complete follow-up rates of *P. falciparum *and *P. vivax *patients**.

In 2009, after the DTMM had been implemented and was fully functioning, there were 534 malaria patients, 285 (53.3%) *P. falciparum*, and 249 (46.7%) *P. vivax*. Compared with the baseline data, the follow-up rates collected by malaria staff on their mobile phones showed significant improvement. The rates for Thai *P. falciparum *and *P. vivax *cases were > 90% (range 98-99%) at first follow-up day (Day 7 or Day 14), to 93% at the last recommended follow-up day (Day 90) (see Figure 5). The rates of follow-up among the migrant patients were lower: 94% on Day 7 to 84% on Day 90 for *P. falciparum*, and 98% on Day 14 to 85% on Day 90 for *P. vivax*.

Self-reported adherence to anti-malarial drug therapy showed high completion rates for *P. falciparum *(94.0%) but fairly low rates for *P. vivax *(42.6%) (see Table 3). Patients' symptoms were captured onto the mobile phones during follow-up visits, either during home visits or at the MC/MP. At 7 days after the first anti-malarial drug dose, a high proportion (83%) of *P. falciparum *patients reported headache, over half (60%) reported muscle pain and fatigue, some (15%) had fever, and about 17% vomited. Similarly at 14 days after the first anti-malarial drug dose, most *P. vivax *patients (72%) reported headache, about half (51%) reported muscle pain and fatigue, and some (12%) had fever (Table 3).

**Table 3:** Malaria case follow-up for *P. falciparum *and *P. vivax *patients, Sai Yok District, 2009

Follow-up On Mobile Phone	*P. falciparum *cases (n = 285)		*P. vivax *cases (n = 249)	
	n	%	n	%
Complete treatment (self-reported)				
No	17	6.0	143	57.4
Yes	268	94.0	106	42.6
Symptoms within the first follow-up day (day 7/day14) post first dose				
Fever	42	14.7	29	11.6
Headache	235	82.5	179	71.9
Chill	10	3.5	6	2.4
Muscle Pain/Fatigue	172	60.4	127	51.0
Vomit	49	17.2	16	6.4
Diarrhea	5	1.8	2	0.8

**Figure 6:**
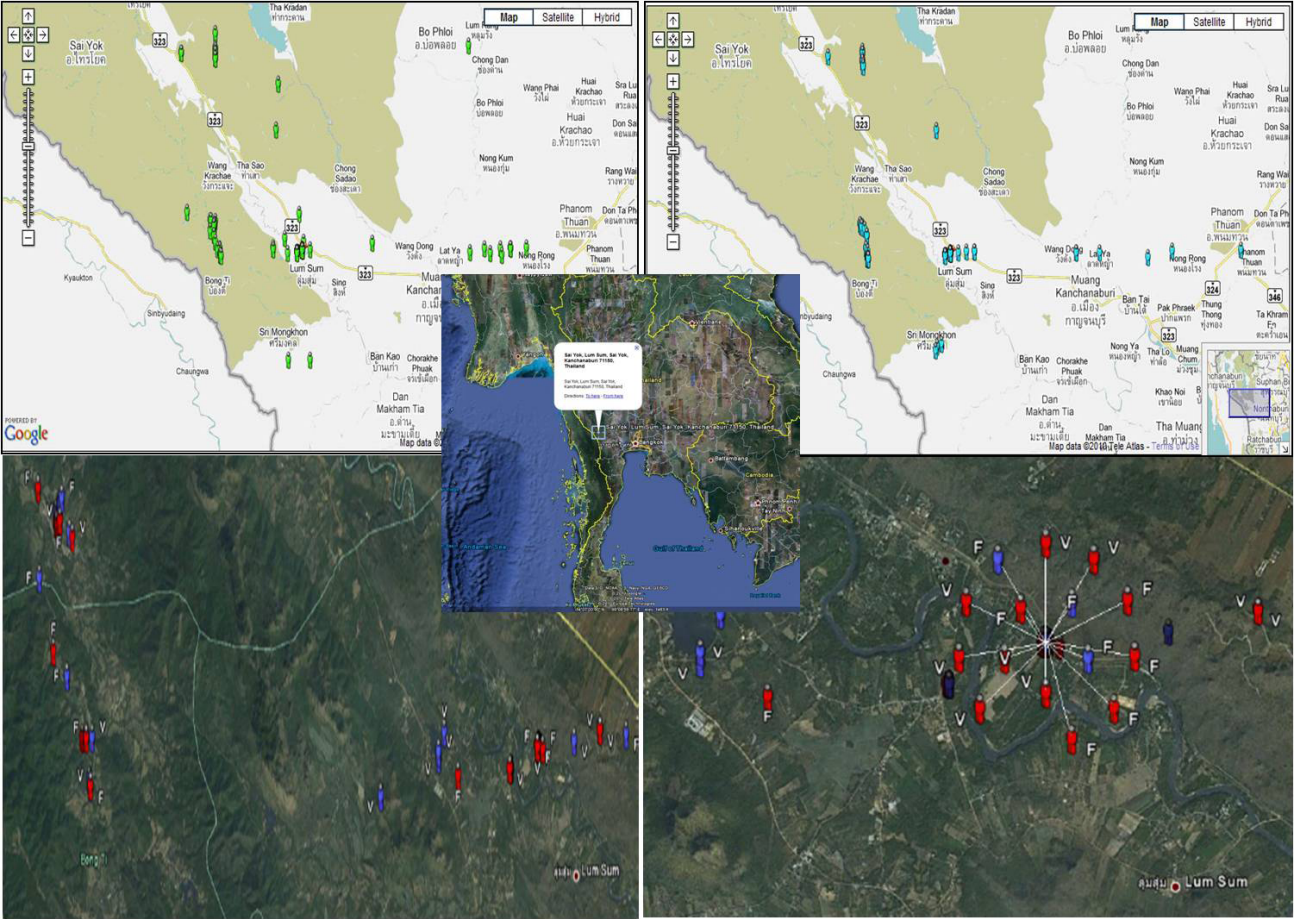
**Screen shots of disease mapping of follow-up cases**.

The data in the DTMM could also be mapped, using a geographical information system (GIS). The geographical locations of cases captured on Day 7 (*P. falciparum*), Day 14 (*P. vivax*) and Day 28 (*P. falciparum *+ *P. vivax*) are shown in Figure 6. No spatial or temporal analysis, as part of the initial version of DTMM, was used in this study. However, the simple disease mapping of these cases at the village location could help malaria staff target preventive action more accurately within the identified high-risk zones. Mapping malaria-risk locations makes it possible to conduct active case management, allocate priorities in control programmes, and plan interventions.

## Discussion

### Malaria cases among border populations

In 2009, in contrast with the previous 5 years, the numbers of malaria cases among Thais in the study areas were higher than among migrant cases who had mostly resided in Thailand ≥ 6 months. The very high numbers of migrant patients in 2008, pre-DTMM, was due to the high cross-border population numbers that particular year. Baseline information during the 5 pre-DTMM years usually showed more *P. falciparum *than *P. vivax *cases; but for the year 2009, the proportion of *P. falciparum*-infected cases was higher among Thais but similar among migrants. Meanwhile, a history of malaria infection was lower among Thais than migrants. These proportions of infection captured from the DTMM reflected disease burden in the areas even though it derived only from symptomatic individuals who came to malaria clinics seeking anti-malarial treatment. Almost all cases in border areas would seek treatment at governmental malaria clinics or the district hospital, since there was no private hospital in the area, and anti-malarial drugs are not available in Thai drug stores. In an epidemiological study conducted in a low-transmission setting on the Thai-Myanmar border [33], it was reported that all *P. falciparum *infections were symptomatic, but approximately 10% of *P. vivax *infections were still asymptomatic. In another cross-sectional study in intense malaria transmission districts near the site of this study, along the Thai-Myanmar border [34], among the surveyed 125 households, 10% were Thai, 42% Mon, and 48% Karen; about 40% of Thai and Karen migrants and almost 30% of Mon migrants reported having suffered from malaria at least once.

The age and sex distribution of the malaria patients reflected the disease situation in the area. Most patients in both groups were working-aged males who laboured in rice and corn farms in the area, while some reported working in forest areas along the border. In a retrospective study along Thailand's borders [35], increasing proportions of malaria cases were shown over 12 years among cross-border migratory foreign workers; these cases were especially concentrated in districts bordering Cambodia and Myanmar. This trend suggested that cross-border seasonal labour might play an important role in malaria transmission. In the other study of factors influencing self-reported malaria among migrants living along the Thai-Myanmar border [34], migrant working conditions played a major role in acquiring malaria.

In contrast with their infection status, most Thai and migrant patients in this study reported that they owned a bed-net and usually slept in a bed-net when they stayed in a transmission area. In a study following 3 years' bed-net use by villagers in Kanchanaburi Province [36], it was reported that conventional insecticide-treated mosquito nets (ITN) had been used over the years by the villagers, but that they needed to be retreated promptly at 6-month intervals, or they would only provide 15% protection against malaria vectors. The results of the current study may reflect the findings of the bed-net study such that even though patients claimed to use bed-nets when they stayed in a transmission area, the ITNs might fail due to being re-treated improperly. A long-term follow-up study on bed-net use [36] also reported that ITN implementation at community level had several technical, operational, economic, and social difficulties, and as a result, low re-treatment rates were observed.

### Adherence to treatment and care

For self-reported adherence to anti-malarial drug therapy, this study found a completion rate of > 90% for *P. falciparum *cases. This markedly rate may due to the short course of treatment; however, this figure was based on self-reports, not direct observations, and may not be completely reliable. In contrast, adherence to treatment among the *P. vivax *cases was < 50%, probably due to the longer treatment course. Similarly, a study of 206 *P. vivax *patients on the northern Thai-Myanmar border [37] found 76% did not complete the full medication course. A study on adherence to treatment among *P. vivax *patients found significant differences in knowledge about malaria and perceptions of the benefits of anti-malarial drugs between adherent and non-adherent groups.

Like any public health programme, the success or failure of a malaria control programme is largely determined by the effective use of the services offered to the public. Monitoring the prompt use of effective drugs would greatly reduce the incidence of severe and complicated disease [22]. Effective, active case monitoring can also increase the chances of capturing relapse and reinfection. In this study, the use of mobile phones, as part of the DTMM, proved effective in increasing case follow-up rates, in line with the days recommended for ideal case-management and monitoring (up to day 90). The follow-up rates for *P. falciparum *and *P. vivax *Thai patients were almost 100% at first follow-up day (Day 7/14), and were maintained at > 90% to Day 90. Even though the follow-up rates for migrant patients appeared lower, at about 85% at Day 90, this could still be considered a marked success. Case follow-up rates using the smartphone in the DTMM were much higher than the five pre-DTMM years. The increases in follow-up rates for migrant workers has made it a promising tool for planning malaria prevention and control programmes in the cross-border multi-drug-resistant malaria containment project.

However, it is possible that the treatment adherence and case-follow-up rates observed in this study may be overestimated. Although the DTMM activities attempted to mimic the routine malaria prevention and control programme situation, efforts to conduct home visits by malaria staff may have been more diligent than normal practices in case management due to sensitizing with the programme and equipment. Nevertheless, this finding reflects what malaria case management could have achieved in terms of promoting adherence to therapy and case monitoring in ideal situation and beyond what will generally be expected for case follow-up to Day 28.

### Monitoring signs and symptoms

During routine follow-up home visits, non-specific symptoms and signs of malaria (fever, headache, shivering/chills, muscle pain/fatigue, diarrhea, and vomiting) were captured on mobile phone. The purpose of this DTMM feature is to monitor the typical symptom of malaria which could be cyclical occurrence and to avoid chance of complicated incident as delay of diagnostic and treatment may lead to severe complications [38]. Other side-effects and severe adverse events (if any) were monitored by malaria staff outside the mobile tool. The information captured by the DTMM smartphone revealed that patient symptoms from first drug dose to first follow-up day were similar to those reported in several other clinical-trial studies [31,39,40].

These symptoms could also have effect on adherence to treatment previously discussed. Drug failure might occur in malaria endemic area by several means including, in part, the patients got relief from major clinical symptoms and thus not adhered to the full course. This could easily go undetected without an effective follow-up system. Propagation of resistant parasites could result in an epidemic without effective case monitoring and the lack of an early warning module [41].

## Conclusions

### DTMM usage

The DTMM has shown successfully integrated into the malaria control programme's operations at the pilot sites. Data in the secured database of the module helped local malaria staff at the MC conduct their activities. The DTMM has succeeded in providing information to local malaria staff for routine case-management activities, and in providing timely summary reports for situational analysis. The DTMM made data collection easier at the local clinic, and additional data were collected on the mobile phone during routine home visits. With more information collected systematically in the database, this module has allowed the exploration of clinical outcomes and the generation of new hypotheses about transmission, and prevention and control measures. It would also be a useful tool for intervention studies, since it can collect reliable and timely variables, and identify cases in study areas. It has the potential to help determine vulnerable predictors of malaria outbreaks.

In summary, malaria case-management using the DTMM appeared to have reached close to the target of national guideline, especially for case-monitoring and follow-up. By using information from the electronic-based data capture and data management with an additional feature of mobile technology for data transfer, follow-up could even be successfully performed beyond day 28. The DTMM has made it easier and faster to collect and process data closer to real-time, compared with the original paper-based process. The module removes repetitive data collection and generates useful reports for malaria control actions. Malaria staff at the pilot site, who used the DTMM, expressed satisfaction with the module's applications. It should be noted, however, that it required several meetings and training sessions to familiarize staff with the DTMM, and to use the smartphone for data collection in areas with and without mobile telephone service. Another issue requiring attention is linkage of the DTMM to the existing healthcare system, making it part of Thailand MOPH's routine work practices. This could be achieved by making the local staff feel ownership and make use of the data collected locally while be able to generate the routine reports needed by MOPH.

### Module operation and management costs

In quantifying the costs of designing, implementing, and maintaining this module, certain factors must be taken into account. The estimates here comprise hardware and software costs, not personnel costs.

The DTMM was developed by BIOPHICS with support from a Microsoft Research Award; its concept and programming parts were posted as open-source. The DTMM requires a server to manage data processing (scheduling follow-up appointments and updating visits) and text message delivery. Currently, the server is located at BIOPHICS, but this can be put as part of the existing computer at VBDU or upper levels. In 2009 figures, the investment cost for the DTMM (minimum for hardware requirements, including server, workstation, and tape back-up) is about US $2,500, and software licenses for Windows and SQL about US $2,500. MC/VBDU staff enter data into the DTMM system using a laptop (or workstation) connected to the internet by an Aircard (maintenance cost about US $20 per month) with unlimited data package. The SMS cost was about US $0.03 per message. At least one low-cost smartphone should be allocated to VBDU staff per operational area; the prices of smartphones have dropped dramatically over the year. In 2009, the DTMM smartphone cost approximately US $300.

### Challenges

The results of this study, and epidemiological data from other studies, show that the prevalence of malaria remains unacceptably high in border areas. The crucial groups requiring attention include hard-to-reach populations, ethnic minorities, and mobile populations. Surveillance data over the years imply that the provinces with the highest incidence rate border Myanmar and Cambodia. Population movements in these areas, together with high drug pressure, are considered responsible for the development and spread of drug-resistant *P. falciparum *malaria [35]. It has been suggested that efforts to isolate tolerant parasites and eliminate malaria require more harmonized and aligned multi-country malaria control and pre-elimination strategies [42].

Thus, in 2008, the WHO and the BVBD of Thailand MOPH developed a programme to contain multi-drug resistant malaria along the Thai-Cambodian border. In this work, recommendations were made to improve malaria treatment adherence by Directly Observed Treatment (DOT), to implement cross-border strategies to harmonize malaria-control strategies, and to share information on drug resistance and follow-up on patients who regularly crossed the border [5,42]. One strategy included an effective surveillance system, which could capture and manage malaria cases in border areas, by incorporating such vulnerable populations [5,6,42]. Therefore, the DTMM was modified and enhanced to be Malaria Information System (MIS) with the purpose to expand for implementation in seven provinces along the Thai-Cambodian border. The modified DTMM (the MIS) is now functioning particularly in response to emergency containment and in planning the elimination of tolerant malaria parasites in the region. The MIS is a collaborative effort between BIOPHICS and the BVBD of Thailand's MOPH, and is funded by WHO and the Bill and Melinda Gates Foundation (BMGF) for 2009-2010 [43].

The MIS, the modified version of the DTMM, is functioning as a surveillance and case management system for malaria incidents at the local level. Like any surveillance system, its value for infectious disease is measured by its ability to provide timely, accurate ''data for action'' to people responsible for effective prevention and control activities, and by its ability to provide ongoing feedback to the primary gatherers of information [44]. It is believed that long-term and accurate datasets, such as that provided by the MIS, are essential for prediction and trend analysis of malaria. In the containment project, the web-based and mobile phone systems have been modified to capture clinical, laboratory, and parasitic outcomes. The enhanced version of the DTMM utilizes more GIS features and spatial analysis which are essential to focus on scarce resources, improve the efficacy of control, and decrease the burden of disease [45]. The modified DTMM also adds spatio-temporal presentations for decision-making and containment purposes. Specifically, within the modified DTMM system launched in the border area, GIS could be useful at both macro and micro levels in planning the provision of health infrastructure, mapping disease distribution, investigating the spatial dynamics of disease transmission, and modeling health service utilization and disease control intervention.

The major challenge for success of the system is data quality, including data integrity, completeness, and timeliness. Since the system is designed to capture data, in part, for migrants and hard-to-reach populations, it should be acknowledged that some malaria infections may not be contracted at the place of reporting. The MIS is in the process of enhancement, to enable it to handle such difficult situations. Dedicated staff were a crucial factor for success of the DTMM; at minimum, they should be well-trained in geographical-information and mobile-technology applications; while this is not difficult, it cannot be over-emphasized. This is an important factor to consider for MIS implementation in new border areas.

The MIS has now been functioning to the eastern Thai-Cambodia border, despite its origins on the western Thai-Myanmar border. Another challenge is to expand the modified version of the DTMM to manage a wider range of endemic areas back on the longer stretching western border. Strengthening MC and VBDU infrastructure and additional financial support are needed in certain locations for sustainability and implementation at a national level. It is a substantial challenge to expand and maintain such a system nationwide and to eventually be part of and managed by the Thailand MOPH malaria surveillance team. Moreover, the system design will need adjustment, so that it is more modular approach, and more easily adapted for use beyond Thailand's borders.

## Competing interests

The authors declare that they have no competing interests. Development of the DTMM was supported by Microsoft Research.

## Authors' contributions

PM, AK, PS, JK were involved in the conceptualization and design of the study, and design of the application module. PK designed and programmed the application module, monitored and maintained the module's implementation, and extracted data for analysis. WS was responsible for managing and supervising the overall malaria-control-programme activities. AK and PM were in charge of monitoring progress of the module application. PM and JK performed statistical analyses and drafted the manuscript. All authors read and approved the final manuscript.
